# Long‐term mortality and cardiovascular events of seven angiotensin receptor blockers in hypertensive patients: Analysis of a national real‐world database: A retrospective cohort study

**DOI:** 10.1002/hsr2.1056

**Published:** 2023-01-31

**Authors:** Wonjae Lee, Jeehoon Kang, Jun‐Bean Park, Won‐Woo Seo, Seung‐Yeon Lee, Woo‐Hyun Lim, Ki‐Hyun Jeon, In‐Chang Hwang, Hack‐Lyoung Kim

**Affiliations:** ^1^ Division of Cardiology, Department of Internal Medicine, Cardiovascular Center Seoul National University Bundang Hospital Seongnam‐si Gyeonggi‐do South Korea; ^2^ Division of Cardiology, Department of Internal Medicine Seoul National University Hospital Seoul South Korea; ^3^ Division of Cardiology, Department of Internal Medicine, Kangdong Sacred Heart Hospital Hallym University Seoul South Korea; ^4^ International Healthcare Center Seoul National University Bundang Hospital Seongnam‐si Gyeonggi‐do South Korea; ^5^ Division of Cardiology, Department of Internal Medicine, Boramae Medical Center Seoul National University College of Medicine Seoul South Korea

**Keywords:** angiotensin receptor blocker, cardiovascular event, hypertension, mortality, prognosis

## Abstract

**Background and Aims:**

Although many angiotensin receptor blockers (ARBs) are widely used, comparative data regarding their impact on clinical outcomes are limited. We aimed to compare the clinical effectiveness of seven ARBs on long‐term cardiovascular outcomes in Korean patients with hypertension.

**Methods:**

Using the Korean National Health Insurance Service database, the data of 780,785 patients with hypertension without cardiovascular disease (CVD) who initiated ARB treatment (candesartan, fimasartan, irbesartan, losartan, olmesartan, telmisartan, or valsartan) in 2014 and underwent this treatment for more than 6 months, were analyzed. Cox‐regression analysis was performed using Losartan as a comparator, as it was the most widely used drug, by adjusting age, sex, diabetes, dyslipidemia, smoking, alcohol drinking, exercise, body mass index, systolic blood pressure, albuminuria, estimated glomerular filtration rate, and concomitant medications. The occurrence of mortality and the rate of major adverse cardiovascular events (MACEs) of the six ARBs was compared with that of losartan.

**Results:**

The median follow‐up duration was 5.94 (interquartile range, 5.87–5.97) years. In the crude analysis of all‐cause mortality and MACEs, fimasartan exhibited the lowest event rates. In the Cox‐regression analysis with adjustment, there was no significant difference in all‐cause mortality among ARBs. The risk of MACEs with ARBs was similar to that with losartan, although the risks with irbesartan (hazard ratio [HR], 1.079; 95% confidence interval [CI], 1.033–1.127; *p* = 0.007) and candesartan (HR: 1.066; 95% CI, 1.028–1.106; *p* = 0.015) were slightly higher.

**Conclusion:**

In a Korean population of patients with hypertension without CVD, six different ARBs showed similar efficacy to losartan in terms of long‐term mortality and MACEs. Further well‐designed prospective studies are required to confirm our findings.

## 1 INTRODUCTION

Angiotensin receptor blockers (ARBs) have a protective effect on the cardiovascular system and effectively lower blood pressure (BP). Additionally, ARBs are well‐tolerated and are recommended as a first‐line choice of antihypertensive medication.^1^, ^2^, ^3^ In particular, ARBs have less common adverse effects, such as dry cough, which has a high incidence in Asian populations treated with angiotensin‐converting enzyme inhibitors (ACEIs).^4^ ARBs are currently the most prescribed antihypertensive drugs in many countries, including South Korea.^2^, ^5^

Currently, nine ARBs are available in the global market. While most ARBs share a common molecular structure which translates into the class effect, each ARB also has a different chemical structure associated with additional benefits.^6^, ^7^, ^8^, ^9^ For example, losartan, candesartan, and valsartan exhibit strong cardiovascular protective effects in patients with heart failure with reduced ejection fraction.^10^, ^11^, ^12^ Valsartan is more beneficial in terms of long‐term cardiovascular prognosis in patients with myocardial infarction.^13^ Owing to their renoprotective effects, losartan and irbesartan have been suggested for the treatment of diabetic nephropathy.^14^, ^15^

In clinical practice, the prevalence of simple hypertension without complications is much higher than that of hypertension with complications.^16^ Therefore, information on the use of ARBs for uncomplicated hypertension is very important. However, there is no evidence concerning which ARBs are the most suitable for patients with hypertension who do not have compelling indications, such as heart failure and myocardial infarction. As ARBs are widely prescribed antihypertensive drugs, a guide for choosing them for patients without cardiovascular disease (CVD) would be valuable to clinicians.

Recently developed ARBs, such as olmesartan and fimasartan, have strong BP‐lowering effects and are widely used in clinical practice. However, evidence of their effectiveness in improving cardiovascular prognosis beyond antihypertensive effect is scarce. Prognostic information associated with the use of these new ARBs will greatly help clinicians treat hypertension.

We conducted a retrospective analysis to compare the effects of different ARB types, including new‐generation ARBs, on long‐term mortality and cardiovascular events in patients with hypertension.

## 2 METHODS

### 2.1 Study patients

The Korean government has operated the National Health Insurance Service (NHIS) to provide medical insurance services to all Korean residents since 1989.^17^ The NHIS has demographic, socioeconomic, and disability registration data to make decisions regarding eligibility and premium charging. Additionally, the NHIS has detailed data on healthcare utilization (procedures, drugs, and other treatments) submitted by medical providers for reimbursement. Using these data, the NHIS established the National Health Information Database (NHID) in 2012 to support public health policies and research.^18^ We used the NHID provided by the NHIS (NHIS‐NHID). This study was conducted after obtaining approval from the Institutional Review Board (IRB) of NHIS (research management number: NHIS‐2020‐1‐110) and Seoul National University Boramae Medical Center (IRB No. GFIRB 2019‐304). As this study was conducted using the database provided by the NHIS, the IRB of Seoul National University Boramae Medical Center waived the need to obtain informed consent. This study was performed in accordance with the relevant guidelines and regulations. Among the individuals included in the NHIS‐NHID from January 1, 2014 to December 31, 2014, a total of 1,336,150 patients with the following inclusion criteria were identified: (1) diagnosis of hypertension (ICD diagnostic code: I10), (2) no history of CVD (ischemic heart disease, stroke, and heart failure), (3) health check‐up in 2014 and presence of major clinical and laboratory data, (4) initiation of ARB administration as an antihypertensive medication in 2014, and (5) ARB administration for >6 months. Administration of other classes of antihypertensive drugs was allowed. Among the nine ARBs marketed worldwide, azilsartan, which was not yet introduced into Korea in 2014, and eprosartan, which has a small number of prescriptions (*n* = 7300), were not included. Further exclusion criteria were as follows: (1) the occurrence of mortality before the date of prescription (*n* = 356) and (2) missing demographic characteristics and examination results (*n* = 547,709). Finally, we analyzed the data of 780,785 patients.

### 2.2 ARBs

The ARBs investigated were candesartan, fimasartan, irbesartan, losartan, olmesartan, telmisartan, and valsartan, according to the Anatomical Therapeutic Chemical codes maintained by World Health Organization (Supporting Information: Table S1).

### 2.3 Collection of clinical variables

Demographic, clinical, and laboratory data were collected from the health check‐up database in 2014. Body mass index (BMI) was calculated by dividing the weight (kg) by the square of height (m^2^). Systolic BP (SBP) and diastolic BP were measured using an oscillometric device in the right upper arm. The measurement was performed three times at the right upper arm, and the average of lower two values were taken, the values reflecting “during drug use.” Cardiovascular risk factors, including diabetes mellitus, dyslipidemia, smoking, alcohol consumption, and household income levels, were obtained using diagnostic codes and questionnaires. After overnight fasting for approximately 12 h, the blood glucose, total cholesterol, high‐density lipoprotein cholesterol, low‐density lipoprotein cholesterol, triglyceride, and creatine levels were measured. Urinalysis was performed and the presence and degree of proteinuria were assessed. The Chronic Kidney Disease Epidemiology Collaboration equation was used to calculate the estimated glomerular filtration rate (eGFR). Considering impacts on cardiovascular outcomes, information on medication of other antihypertensive agents (calcium channel blockers, beta‐blockers, ACEIs, and diuretics), antithrombotic agents, and statins was also obtained in cases where the medication was prescribed for more than 6 months at the time of initial ARB prescription.

### 2.4 Clinical outcomes

All‐cause mortality and major adverse cardiovascular events (MACEs) were the main outcome variables in this study. All‐cause mortality was determined from the date of death. MACEs were defined as cardiac death, nonfatal myocardial infarction, stroke, hospitalization for heart failure, and coronary revascularization. The Korean Standard Classification of Diseases (KCD‐7‐based ICD‐10) was used to define each MACE (Supporting Information: Table S2). The first date when the above ICD‐10 codes were present in the claims data was defined as the event date.

### 2.5 Statistical analysis

The *χ* ^2^ test for categorical variables and the Kruskal–Wallis test for continuous variables were performed to evaluate the differences in the distribution of demographic characteristics, incidence of MACEs, and distribution of all‐cause mortality depending on the type of ARB. Hazard ratios (HR) and 95% confidence intervals (CI) of all‐cause mortality and MACEs were calculated by using a Cox proportional‐hazard model adjusted for age, sex, diabetes (E10–E14), dyslipidemia (E78), smoking, alcohol drinking, exercise, household income, BMI, SBP, eGFR, and concomitant medications, including calcium antagonists, beta‐blockers, ACEIs, diuretics, antithrombotic agents, and statins. The proportional assumption of the Cox analysis was conducted for Cox proportional‐hazard modeling. In univariate analysis, a log‐rank test was conducted. Subsequently, multiple analyses were conducted using the Cox proportional hazard model. All statistical analyses were performed using SAS 9.4 software (SAS Institute). All two‐sided *p* < 0.05 were considered statistically significant.

## 3 RESULTS

Table 1 shows the distribution of demographic and clinical characteristics according to ARB type of study patients. A total of 58,892 (7.5%), 21,041 (2.7%), 37,170 (4.8%), 298,461 (38.2%), 100,348 (12.9%), 125,730 (16.1%), and 139,143 (17.8%) patients were taking candesartan, fimasartan, irbesartan, losartan, olmesartan, telmisartan, and valsartan, respectively. The study included 442,694 (56.7%) men and 338,091 (43.3%) women (average age, 59.62 years). The average age was the highest in the losartan group (60.75 years) and the lowest in the fimasartan group (57.66 years). The proportion of women was the highest in the losartan group (46.2%) and the lowest in the telmisartan group (39.4%). The average SBP of all study patients was 129.11 mmHg. The average SBP was the highest in the valsartan group (130.17 mmHg) and the lowest in the fimasartan group (127.37 mmHg). The prevalence of diabetes mellitus in all patients was 24.4%. The irbesartan group had the highest prevalence of diabetes mellitus (31.0%) while the fimasartan group had the lowest (21.1%). The prevalence of dyslipidemia in all patients was 38.2%, with the candesartan group exhibiting the highest prevalence (45.4%) and the losartan group exhibiting the lowest (35.4%). The proportion of smokers and drinkers was the highest in the telmisartan group and the lowest in the irbesartan group. The proportion of patients taking calcium antagonists, beta‐blockers, ACEIs, diuretics, antithrombotic agents, and statins was 22.5%, 8.8%, 0.5%, 6.1%, 26.2%, and 30.6%, respectively.

**Table 1:** Baseline clinical characteristics of study patients

Characteristic	Total (*n* = 780,785)		Candesartan (*n* = 58,892)		Fimasartan (*n* = 21,041)		Irbesartan (*n* = 37,170)		Losartan (*n* = 298,461)		Olmesartan (*n* = 100,348)		Telmisartan (*n* = 125,730)		Valsartan (*n* = 139,143)		*p* value
Age (years), median (Q1, Q3)	59.0 (52.0, 67.0)		60.0 (53.0, 68.0)		57.0 (50.0, 65.0)		60.0 (54.0, 68.0)		60.0 (54.0, 68.0)		58.0 (52.0, 66.0)		58.0 (52.0, 66.0)		58.0 (52.0, 66.0)		<0.001^a^
Sex																	<0.001*
Male, *n* (%)	442,694	56.7	32,448	55.1	11,819	56.2	20,220	54.4	160,732	53.9	58,953	58.8	76,198	60.6	82,324	59.2	
Female, *n* (%)	338,091	43.3	26,444	44.9	9,222	43.8	16,950	45.6	137,729	46.2	41,395	41.3	49,532	39.4	56,819	40.8	
BMI (kg/m²), median (Q1, Q3)	25.2 (23.2, 27.3)		25.1 (23.2, 27.1)		25.1 (23.2, 27.2)		25.1 (23.2, 27.1)		25.0 (23.1, 27.1)		25.4 (23.5, 27.5)		25.3 (23.4, 27.4)		25.2 (23.3, 27.4)		<0.001^a^
SBP (mmHg), median (Q1, Q3)	130.0 (120.0, 138.0)		129.0 (120.0, 137.0)		128.0 (119.0, 136.0)		130.0 (120.0, 138.0)		130.0 (120.0, 139.0)		128.0 (120.0, 136.0)		129.0 (120.0, 137.0)		130.0 (120.0, 139.0)		<0.001^a^
DBP (mmHg), median (Q1, Q3)	80.0 (72.0, 85.0)		80.0 (70.0, 84.0)		80.0 (71.0, 85.0)		80.0 (71.0, 85.0)		80.0 (72.0, 85.0)		80.0 (70.0, 84.0)		80.0 (71.0, 85.0)		80.0 (73.0, 86.0)		<0.001^a^
Diabetes (yes), *n* (%)	190,528	24.4	16,370	27.8	4,435	21.1	11,532	31.0	71,122	23.8	23,475	23.4	29,727	23.6	33,867	24.3	<0.001*
Dyslipidemia (Yes), *n* (%)	398,485	38.2	26,728	45.4	8,608	40.9	16,616	44.7	105,665	35.4	36,678	36.6	47,678	37.9	56,512	40.6	<0.001*
Smoke (yes), *n* (%)	143,226	18.3	9,701	16.5	3,946	18.8	5,828	15.7	51,707	17.3	19,598	19.5	24,918	19.8	27,528	19.8	<0.001*
Drink (yes), *n* (%)	233,103	29.9	15,814	26.9	6,370	30.3	9,860	26.5	83,784	28.1	32,402	32.3	40,831	32.5	44,042	31.7	<0.001*
Physical activity (yes), *n* (%)	287,360	36.8	21,311	36.2	7,852	37.3	13,193	35.5	111,244	37.3	36,712	36.6	45,923	36.5	51,125	36.7	<0.001*
Income quartiles																	<0.001*
Lowest, *n* (%)	158,555	20.3	11,338	19.3	4,335	20.6	7,182	19.3	63,322	21.2	20,103	20.0	24,845	19.8	27,430	19.7	
Second, *n* (%)	141,405	18.1	9,910	16.8	3,883	18.5	6,184	16.6	55,782	18.7	18,126	18.1	22,701	18.1	24,819	17.8	
Third, *n* (%)	185,401	23.8	13,419	22.8	5,100	24.2	8,501	22.9	71,642	24.0	23,985	23.9	29,478	23.5	33,276	23.9	
Highest, *n* (%)	295,424	37.8	24,225	41.1	7,723	36.7	15,303	41.2	107,715	36.1	38,134	38.0	48,706	38.7	53,618	38.5	
FBS (mg/dl), median (Q1, Q3)	101.0 (92.0, 116.0)		102.0 (92.0, 116.0)		100.0 (91.0, 113.0)		102.0 (93.0, 119.0)		101.0 (92.0, 116.0)		102.0 (92.0, 116.0)		102.0 (93.0, 116.0)		101.0 (92.0, 116.0)		<0.001^a^
Total cholesterol (mg/dl), median (Q1, Q3)	186.0 (163.0, 212.0)		182.0 (158.0, 208.0)		186.0 (163.0, 211.0)		182.0 (158.0, 207.0)		187.0 (164.0, 212.0)		188.0 (164.0, 214.0)		188.0 (164.0, 213.0)		185.0 (161.0, 211.0)		<0.001^a^
HDL (mg/dl), median (Q1, Q3)	50.0 (43.0, 60.0)		50.0 (43.0, 60.0)		51.0 (43.0, 60.0)		50.0 (42.0, 59.0)		51.0 (43.0, 60.0)		50.0 (43.0, 59.0)		50.0 (42.0, 59.0)		50.0 (43.0, 60.0)		<0.001^a^
LDL (mg/dl), median (Q1, Q3)	106.0 (84.0, 129.0)		102.0 (80.0, 126.0)		105.0 (83.0, 128.0)		102.0 (81.0, 125.0)		106.0 (85.0, 130.0)		107.0 (84.0, 131.0)		107.0 (84.0, 131.0)		104.0 (82.0, 128.0)		<0.001^a^
TG (mg/dl), median (Q1, Q3)	124.0 (88.0, 179.0)		122.0 (87.0, 174.0)		123.0 (87.0, 178.0)		121.0 (86.0, 173.0)		124.0 (88.0, 177.0)		128.0 (90.0, 184.0)		125.0 (89.0, 180.0)		125.0 (88.0, 180.0)		<0.001^a^
GFR (ml/min), median (Q1, Q3)	81.0 (70.0, 94.0)		81.0 (69.0, 94.0)		82.0 (71.0, 96.0)		80.0 (68.0, 93.0)		81.0 (69.0, 93.0)		81.0 (70.0, 94.0)		82.0 (71.0, 96.0)		82.0 (71.0, 95.0)		<0.001^a^
Proteinuria																	<0.001*
Negative (−), *n* (%)	725,690	92.9	54,556	92.6	19,946	94.8	33,643	90.5	278,075	93.2	93,991	93.7	117,165	93.2	128,314	92.2	
Trace (±), *n* (%)	23,719	3.0	1880	3.2	520	2.5	1312	3.5	8901	3.0	2859	2.9	3707	3.0	4540	3.3	
Positive (+1), *n* (%)	18,836	2.4	1,448	2.5	366	1.7	1182	3.2	7136	2.4	2148	2.1	2848	2.3	3708	2.7	
Positive (+2), *n* (%)	9162	1.2	744	1.3	162	0.8	705	1.9	3251	1.1	967	1.0	1455	1.2	1878	1.4	
Positive (+3), *n* (%)	2783	0.36	219	0.37	39	0.19	263	0.71	908	0.30	322	0.32	462	0.37	570	0.41	
Positive (+4), *n* (%)	595	0.08	45	0.08	8	0.04	65	0.17	190	0.06	61	0.06	93	0.07	133	0.10	
Calcium channel blockers (yes), *n* (%)	175,253	22.5	16,224	27.6	4219	20.1	11,023	29.7	80,508	27.0	18,771	18.7	18,162	14.5	26,346	18.9	<0.001*
Beta‐blockers (yes), *n* (%)	68,705	8.8	6245	10.6	1288	6.1	3904	10.5	24,380	8.2	8044	8.0	10,233	8.1	14,611	10.5	<0.001*
ACEIs (yes), *n* (%)	4060	0.52	341	0.58	142	0.67	237	0.64	1280	0.43	474	0.47	592	0.47	994	0.71	<0.001*
Diuretics (yes), *n* (%)	47,779	6.1	3302	5.6	1485	7.1	2046	5.5	17,074	5.7	5616	5.6	8070	6.4	10,186	7.3	<0.001*
Antithrombotic agents (yes), *n* (%)	204,531	26.2	16,825	28.6	4,270	20.3	11,593	31.2	78,708	26.4	26,611	26.5	30,908	24.6	35,616	25.6	<0.001*
Statins (yes), *n* (%)	239,239	30.6	22,323	37.9	6725	32.0	13,422	36.1	84,854	28.4	28,848	28.8	37,632	29.9	45,435	32.7	<0.001*

Abbreviations: ACEI, angiotensin‐converting enzyme inhibitor; BMI, body mass index; DBP, diastolic blood pressure; FBS, fasting blood sugar; GFR, glomerular filtration rate; HDL, high‐density lipoprotein; LDL, low‐density lipoprotein; SBP, systolic blood pressure; TG, triglyceride.

^a^ *p* value was obtained using *χ* ^2^ test.

* *p* value was obtained using the Kruskal–Wallis test.

The incidence of all‐cause mortality and MACEs is presented in Table 2. During the median follow‐up period of 5.94 (interquartile range, 5.87–5.97) years, the all‐cause mortality and MACE rates of the study patients were 2.9% and 5.4%, respectively. The all‐cause mortality rate was the highest in the losartan group (3.2%) and the lowest in the fimasartan group (2.3%). The incidence of MACEs was the highest in the irbesartan group (6.17%) and the lowest in the fimasartan group (4.6%). A similar trend was observed with the individual MACEs; the incidence rate was the highest in the irbesartan group (6.2%) and the lowest in the fimasartan group.

**Table 2:** Incidence of all‐cause mortality and MACE

	Total (*n* = 780,785)		Candesartan (*n* = 58,892)		Fimasartan (*n* = 21,041)		Irbesartan (*n* = 37,170)		Losartan (*n* = 298,461)		Olmesartan (*n* = 100,348)		Telmisartan (*n* = 125,730)		Valsartan (*n* = 139,143)		*p* value*
	*n*	%	*n*	%	*n*	%	*n*	%	*n*	%	*n*	%	*n*	%	*n*	%	
All‐cause mortality	22,291	2.9	1701	2.9	473	2.3	1080	2.9	9566	3.2	2619	2.6	3114	2.5	3738	2.7	<0.001
MACE	42,216	5.4	3445	5.9	961	4.6	2293	6.2	16,932	5.7	5119	5.1	6159	4.9	7307	5.3	<0.001
Cardiac death	4121	0.53	325	0.55	76	0.36	236	0.63	1750	0.59	447	0.45	562	0.45	725	0.52	<0.001
**Nonfatal** MI	7501	0.96	608	1.0	178	0.85	379	1.0	2958	0.99	928	0.92	1154	0.92	1296	0.93	0.025
Stroke	27,900	3.6	2230	3.8	631	3.0	1531	4.1	11,265	3.8	3327	3.3	4076	3.2	4840	3.5	<0.001
HF	8335	1.1	739	1.3	200	1.0	479	1.3	3319	1.1	1060	1.1	1141	0.91	1397	1.0	<0.001
Coronary revascularization	150	0.02	11	0.02	3	0.01	6	0.02	74	0.02	20	0.02	16	0.01	20	0.01	0.189

Abbreviations: HF, heart failure; MACE, major adverse cardiovascular event; MI, myocardial infarction.

* *p* value was obtained using the *χ* ^2^ test.

The risks for all‐cause mortality and MACEs according to different ARBs, compared with those of losartan, are presented in Table 3. In the crude model, the HRs were lower with values of 0.901, 0.701, 0.905, 0.813, 0.771, and 0.838 in the candesartan, fimasartan, irbesartan, olmesartan, telmisartan, and valsartan groups, respectively. For MACEs, the candesartan and irbesartan groups showed a higher risk with HR of 1.039 and 1.098, while fimasartan, olmesartan, telmisartan, and valsartan showed a lower risk than that of the losartan group in the crude model with values of 0.810, 0.901, 0.864, and 0.932, respectively. In the adjusted model, all‐cause mortality risks were similar among all ARBs. Although not substantial, the HRs of MACEs were 1.066 (95% CI, 1.028–1.106; *p* = 0.015) and 1.079 (95% CI, 1.033–1.127; *p* = 0.007) in the candesartan and irbesartan groups, respectively, compared with HR of the losartan group. There was no significant difference in the risk of MACEs among other ARBs. The results of the analysis of individual MACE are presented in Supporting Information: Table S3.

**Table 3:** Risks for all‐cause mortality and MACE according to different ARBs compared to those associated with losartan

	Crude model				Adjusted model			
	HR	95% CI		*p* value	HR	95% CI		*p* value
		Low	High			Low	High	
*All‐cause mortality (ref*. = *losartan)*								
Candesartan	0.901	0.856	0.948	0.003	0.994	0.943	1.046	0.613
Fimasartan	0.701	0.639	0.769	<0.001	0.975	0.889	1.070	0.858
Irbesartan	0.905	0.850	0.964	0.009	0.947	0.889	1.008	0.180
Olmesartan	0.813	0.778	0.849	0.065	1.004	0.961	1.049	0.256
Telmisartan	0.771	0.740	0.802	<0.001	0.965	0.927	1.005	0.331
Valsartan	0.838	0.807	0.870	0.781	0.993	0.956	1.031	0.538
*MACE (ref*. = *losartan)*								
Candesartan	1.039	1.002	1.078	<0.001	1.066	1.028	1.106	0.015
Fimasartan	0.810	0.759	0.864	<0.001	1.001	0.938	1.069	0.400
Irbesartan	1.098	1.051	1.146	<0.001	1.079	1.033	1.127	0.007
Olmesartan	0.901	0.873	0.930	0.001	1.017	0.986	1.050	0.567
Telmisartan	0.864	0.839	0.890	<0.001	0.995	0.966	1.024	0.019
Valsartan	0.932	0.907	0.958	0.262	1.022	0.994	1.050	0.771

*Note*: The following variables were controlled in the adjusted model: age, sex, body mass index, systolic blood pressure, diabetes mellitus, dyslipidemia, cigarette smoking, alcohol drinking, physical activity, household income, glomerular filtration rate, and the use of calcium channel blockers, beta‐blockers, angiotensin‐converting enzyme inhibitors, diuretics, antithrombotics, and statins.

Abbreviations: ARB, angiotensin receptor blocker; CI, confidence interval; HR, hazard ratio; MACE, major adverse cardiovascular event.

Subgroup analyses were conducted according to age, sex, and diabetes status (Supporting Information: Table S4). The age subgroups included patients aged ≥ 70 years and those aged < 70 years. Several ARBs had different HRs for all‐cause mortality and MACEs in the crude model, but there was no substantial difference in the adjusted model, which was similar to the results of the overall study population. We conducted stratified analysis with only ARB, ARB with one class of antihypertensive drug, and ARB with two or more classes of antihypertensive drugs (Supporting Information: Table S5). In the analysis including only the ARB group for MACEs, the HR of the candesartan group was 1.256 (95% CI, 1.035–1.524; *p* = 0.109) and that of the irbesartan group was 1.238 (95% CI, 0.978–1.569; *p* = 0.241). There was no substantial difference in all‐cause mortality and MACEs in other groups.

## 4 DISCUSSION

In this analysis of the real‐world data of a large Korean population of patients with hypertension without CVD, seven different ARBs had similar mortality rates during a median follow‐up period of 5.94 years. Our subgroup analysis of geriatric patients (aged >70 years) and patients with diabetes also demonstrated no substantial differences among ARBs. Although some ARBs showed better effects than those of losartan in terms of MACE occurrence, the degree of difference was only marginal and, thus, less likely to be clinically important. We also showed that newer ARBs, such as fimasartan and olmesartan, had a cardiovascular prognosis similar to that of other ARBs. To our knowledge, this is the first study to compare the efficacy of seven different ARBs on long‐term clinical outcomes in patients with hypertension. In particular, the results of our study are noteworthy as they also provide data on the long‐term prognostic effects of fimasartan and olmesartan, which have scarcely been reported.

Most of the data from existing studies comparing ARBs relate to the short‐term efficacy of the BP‐lowering effect or pleiotropic effect of ARBs.^19^, ^20^, ^21^, ^22^, ^23^, ^24^ Few studies have compared the long‐term clinical outcomes of different types of ARBs. A study of 6876 geriatric patients with heart failure compared the effects of five ARBs (candesartan, irbesartan, losartan, telmisartan, and valsartan) and found that losartan was associated with a lower survival rate than that of other ARBs.^25^ Antoniou et al.^26^ investigated 54,186 patients with diabetes and showed that those taking telmisartan and valsartan were associated with a lower risk of development of acute myocardial infarction, heart failure, and stroke than those taking irbesartan, losartan, and candesartan. Our study differs from these two studies in that we included patients with hypertension and also showed the effectiveness of the new ARBs, fimasartan, and olmesartan. Therefore, our findings are applicable to a broader population. One observational study from Canada showed that patients with hypertension treated with irbesartan had the lowest rate of developing cardiovascular events compared with those receiving other ARBs.^27^ However, the primary goal of this study was to compare the effects of ARBs with other antihypertensive drug classes, and only four ARBs (candesartan, irbesartan, losartan, and valsartan) were analyzed in this sthsrudy. Moreover, the study population taking ARBs was relatively small (*n* = 3490). A study in Taiwan that analyzed a large number of patients (*n* = 690,463) from claims data compared the effects of six ARBs (candesartan, irbesartan, losartan, olmesartan, telmisartan, and valsartan) and found that olmesartan did not increase long‐term cardiovascular risk compared with that by losartan.^28^ This study deserves attention in that it elucidates the effect of olmesartan, unlike previous studies. However, the study population was more heterogeneous as patients with underlying CVD were not excluded, contrary to the case in our study.

ARBs introduced in the early phase have been used in many large‐scale randomized clinical trials (RCTs) with long‐term follow‐up. Most of these studies were designed to compare other classes of antihypertensive drugs, which led to compelling indications for various medical conditions.^11^, ^29^, ^30^, ^31^ Validating the effectiveness of certain drugs with a well‐designed study is important for clinicians prescribing the drug. However, it is difficult to conduct large‐scale RCTs on newly developed ARBs because many ARBs supported by strong evidence are already available. In this situation, retrospective analyses using real‐world data may be helpful. In particular, claims data enable analysis of the data of many patients. Newer ARBs, including olmesartan, fimasartan, and azilsartan, are known to have higher potency compared with previous ARBs.^32^, ^33^ Many head‐to‐head studies have revealed more potent antihypertensive effects, particularly when compared with losartan or valsartan.^22^, ^34^, ^35^ However, these newer ARBs do not have compelling evidence of long‐term cardiovascular outcomes, and the clinical use of these drugs is limited. Our study demonstrates that newer ARBs have similar long‐term clinical outcomes to those of earlier ARBs, based on the data of many patients in the real world. We believe that these data will help clinicians use new ARBs.

Most ARBs share a common molecular structure, which contributes to the class effect of ARBs.^36^ However, ARBs have considerable differences in their molecular structures, which can show differences in their clinical benefits. For example, the number of hydrogen bonds that affect binding affinity is different among ARBs, and this can translate into various antihypertensive effects.^37^ Several studies comparing the antihypertensive effect of ARBs have shown differences in the magnitude of BP reduction.^34^, ^35^ This difference in BP control may affect patients' prognoses. In patients with acute myocardial infarction, comparative analysis revealed that insurmountable ARBs, which have a longer and more stable duration of action, were more effective on long‐term clinical outcomes compared with surmountable ARBs.^38^ A study with a Swedish heart failure registry showed that patients taking insurmountable ARB (candesartan) had better all‐cause mortality rates compared with surmountable ARB (losartan).^38^ Unlike these studies, our results revealed no significant differences in cardiovascular outcomes of six ARBs compared with those of losartan, suggesting that the difference of ARBs is not clinically relevant in patients without CVD. Further studies are warranted to assess whether the class effect applies to certain diseases.

This study had several limitations. First, it included patients receiving other classes of antihypertensive medications, in addition to ARBs. Although the concomitant use of other antihypertensive medications was adjusted for the multivariable analysis, we also conducted stratified analysis. Some ARBs showed worse outcomes than those of others in the analysis of the ARB group. We could not find clinically meaningful explanations for the observed difference, which was also not consistent with the findings of previous studies. Second, although we included patients receiving ARBs for more than 6 months, the on‐treatment duration might have varied among ARBs. Third, the characteristics of the seven ARBs differ, reflecting current practice in that certain ARBs are used for compelling indications and there is preference for a stronger agent in younger patients. These may partly explain the difference in the crude and adjusted models. Fourth, several variables, such as BP and BMI, have a dynamic nature. Although the measurement of these variables and the initiation of ARBs were performed in the same year, the timing does not exactly match and the dynamic nature could not be reflected. Especially, BP could not reflect the efficacy of each ARB, thus, limiting interpretation of our results. Finally, as this is a real‐world study with a large sample size, the interpretation of the study findings should not depend solely on the *p* value. Thus, we considered the sample size, 95% CI, and clinical significance of the observed effect when interpreting the results.

## 5 CONCLUSIONS

In this study, which targeted a large number of Korean adults without CVD using national health data, we found that six ARBs (candesartan, fimasartan, irbesartan, losartan, olmesartan, telmisartan, and valsartan) had comparable effects in terms of long‐term cardiovascular risk compared with that of losartan. These findings also suggest that fimasartan and olmesartan, newly developed strong ARBs, may also have similar cardiovascular effects compared to those of earlier traditional ARBs. Better‐designed prospective studies are needed to confirm our findings.

## AUTHOR CONTRIBUTIONS

**Wonjae Lee**: Data curation; formal analysis; investigation; methodology; project administration; validation; writing – original draft; writing – review and editing. **Jeehoon Kang**: Investigation; methodology; supervision; validation; writing – review and editing. **Jun‐Bean Park**: Investigation; methodology; supervision; validation; writing – review and editing. **Won‐Woo Seo**: Investigation; methodology; supervision; validation; writing – review and editing. **Seung‐Yeon Lee**: Validation; writing – original draft. **Woo‐Hyun Lim**: Investigation; methodology; supervision; validation; writing – review and editing. **Ki‐Hyun Jeon**: Investigation; methodology; supervision; validation; writing – review and editing. **In‐Chang Hwang**: Investigation; methodology; supervision; validation; writing – review and editing. **Hack‐Lyoung Kim**: Conceptualization; data curation; formal analysis; investigation; methodology; project administration; supervision; validation; writing – review and editing.

## CONFLICT OF INTEREST

The authors declare that the research was conducted in the absence of any commercial or financial relationships that could be construed as a potential conflict of interest.

## ETHICS STATEMENT

This study was approved by the IRB of NHIS (research management number: NHIS‐2020‐1‐110) and Seoul National University Boramae Medical Center (IRB No. GFIRB 2019‐304).

## TRANSPARENCY STATEMENT

The lead author Hack‐Lyoung Kim affirms that this manuscript is an honest, accurate, and transparent account of the study being reported; that no important aspects of the study have been omitted; and that any discrepancies from the study as planned (and, if relevant, registered) have been explained.

## Supporting information

Supporting information.

## Data Availability

The data that support the findings of this study are available from National Health Information Database provided by the National Health Insurance Service. Restrictions apply to the availability of these data, which were used under a policy of the NHIS. The data and materials other than the raw data underlying the study are available from the corresponding author, Dr. Hack‐Lyoung Kim, upon reasonable request.
