# Supplementary material for: Long‐term mortality and cardiovascular events of seven angiotensin receptor blockers in hypertensive patients: Analysis of a national real‐world database: A retrospective cohort study
Source: Health Sci Rep. 2023 Jan 31;6(2):e1056. doi: 10.1002/hsr2.1056 (PMC9889622; doi:10.1002/hsr2.1056)
Supplement: Supplementary file 1 — Supporting information. [file HSR2-6-e1056-s001.pdf]

Supplementary Table S1. Definitions of drugs analyzed

ARB type	ATC code
**Candesartan**	C09CA06, C09DB07, C09DA06
**Fimasartan**	C09CA10, C09DA10
**Irbesartan**	C09CA04, C09DA04
**Losartan**	C09CA01, C09DA01, C09DB06
**Olmesartan**	C09CA08, C09DB02, C09DA08, C09DX03, C10BX
**Telmisartan**	C09CA07, C09DA07, C09DB04
**Valsartan**	C09CA03, C09DB01, C09DA03, C09DB08, C09DX04

ARB, angiotensin receptor blocker; ATC, anatomical therapeutic chemical.

Supplementary Table S2. Definitions of clinical events

Clinical event	KCD code	Definition
**MACE**	I21, I22, I60 - I64, I50, I110, I130, I132, O1640, O1641, O1648, O1649, OA641, OA647, OA648, OA649, I46	At least one of these codes.
**Cardiac death**	I110, I21, I130, I132, I46, I50, I61 – I64	Death record with at least one of these codes.
**Non-fatal MI**	I21-I22	At least one of these codes.
**Stroke**	I60-I64	At least one of these codes.
**Hospitalization for HF**	I110, I130, I132, I50	At least one of these codes and record of admission.
**Coronary revascularization**	O1640, O1641, O1648, O1649, OA640, OA641, OA647, OA648, OA649	At least one of these codes.

MACE, major adverse cardiovascular event; MI, myocardial infarction; HF, heart failure.

Supplementary Table S3. Results of Cox-regression analyses showing the hazard ratios of each component of MACE according to different ARBs

Cardiac death (Ref.=Losartan)	Crude model				Adjusted model*			
	HR	95% CI		P-value	HR	95% CI		P-value
		Low	High			Low	High	
**Candesartan**	0.951	0.845	1.071	0.409	1.027	0.912	1.157	0.656
**Fimasartan**	0.628	0.499	0.789	<0.001	0.897	0.713	1.129	0.355
**Irbesartan**	1.094	0.955	1.253	0.197	1.119	0.976	1.282	0.107
**Olmesartan**	0.765	0.690	0.849	<0.001	0.949	0.855	1.053	0.327
**Telmisartan**	0.767	0.698	0.844	<0.001	0.977	0.888	1.076	0.638
**Valsartan**	0.900	0.826	0.982	0.018	1.066	0.977	1.163	0.150

Non-fatal MI (Ref.=Losartan)	Crude model				Adjusted model*			
	HR	95% CI		P-value	HR	95% CI		P-value
		Low	High			Low	High	
**Candesartan**	1.049	0.961	1.145	0.283	1.045	0.957	1.141	0.324
**Fimasartan**	0.863	0.741	1.003	0.055	0.999	0.858	1.162	0.988
**Irbesartan**	1.035	0.930	1.152	0.529	0.996	0.895	1.109	0.941
**Olmesartan**	0.937	0.871	1.009	0.085	1.001	0.930	1.078	0.971
**Telmisartan**	0.930	0.869	0.995	0.036	1.004	0.938	1.076	0.905
**Valsartan**	0.947	0.887	1.011	0.104	0.988	0.925	1.055	0.716

Stroke (Ref.=Losartan)	Crude model				Adjusted model*			
	HR	95% CI		P-value	HR	95% CI		P-value
		Low	High			Low	High	
**Candesartan**	1.009	0.964	1.056	0.690	1.042	0.996	1.091	0.077
**Fimasartan**	0.800	0.738	0.867	<0.001	0.997	0.920	1.081	0.946
**Irbesartan**	1.100	1.043	1.160	0.001	1.088	1.031	1.147	0.002
**Olmesartan**	0.881	0.847	0.915	<0.001	1.004	0.965	1.043	0.859
**Telmisartan**	0.860	0.830	0.891	<0.001	1.004	0.968	1.041	0.831
**Valsartan**	0.927	0.897	0.959	<0.001	1.029	0.995	1.065	0.095

HF (Ref.=Losartan)	Crude model				Adjusted model*			
	HR	95% CI		P-value	HR	95% CI		P-value
		Low	High			Low	High	
**Candesartan**	1.139	1.052	1.234	0.001	1.166	1.076	1.263	<0.001

**Fimasartan**	0.867	0.751	1.000	0.049	1.097	0.951	1.265	0.206
**Irbesartan**	1.169	1.062	1.286	0.001	1.136	1.032	1.251	0.009
**Olmesartan**	0.955	0.892	1.024	0.196	1.093	1.019	1.171	0.012
**Telmisartan**	0.820	0.767	0.877	<0.001	0.960	0.897	1.027	0.232
**Valsartan**	0.912	0.857	0.971	0.004	1.003	0.942	1.068	0.927

**Coronary revascularization (Ref.=Losartan)**	**Crude model**				**Adjusted model***			
	HR	95% CI		P-value	HR	95% CI		P-value
		Low	High			Low	High	
**Candesartan**	0.770	0.409	1.450	0.418	0.687	0.363	1.298	0.248
**Fimasartan**	0.595	0.188	1.888	0.378	0.697	0.219	2.215	0.541
**Irbesartan**	0.663	0.289	1.524	0.333	0.561	0.244	1.293	0.175
**Olmesartan**	0.816	0.498	1.337	0.420	0.880	0.536	1.447	0.615
**Telmisartan**	0.521	0.303	0.894	0.018	0.566	0.329	0.976	0.041
**Valsartan**	0.595	0.363	0.975	0.039	0.598	0.363	0.984	0.043

*The following variables were adjusted: age, sex, diabetes mellitus, dyslipidemia, smoking, drinking, exercise, body mass index, systolic blood pressure, albuminuria, glomerular filtration rate, and concomitant medications including calcium antagonists, beta-blockers, angiotensin converting enzyme inhibitor, diuretics, antithrombotic agents, and statins.

CI, confidence interval; HR, hazard ratio.

Supplementary Table S4-1. Results of Cox-regression analyses showing the hazard ratios of mortality and MACE according to different ARBs in patients ≥70 years old (n=154,427)

All-cause mortality (Ref.=Losartan)	Crude model				Adjusted model*				Adjusted model (with age variable)			
	HR	95% CI		P-value	HR	95% CI		P-value	HR	95% CI		P-value
		Low	High			Low	High			Low	High	
**Candesartan**	0.956	0.894	1.022	0.598	0.975	0.911	1.043	0.755	0.990	0.925	1.059	0.930
**Fimasartan**	0.955	0.844	1.080	0.791	1.006	0.889	1.138	0.447	1.058	0.935	1.197	0.232
**Irbesartan**	0.888	0.818	0.965	0.111	0.892	0.821	0.969	0.028	0.940	0.865	1.021	0.133
**Olmesartan**	0.961	0.908	1.018	0.403	0.994	0.939	1.053	0.257	1.028	0.971	1.089	0.170
**Telmisartan**	0.906	0.858	0.956	0.119	0.942	0.892	0.995	0.316	0.962	0.910	1.016	0.201
**Valsartan**	0.926	0.881	0.974	0.485	0.957	0.909	1.006	0.676	0.975	0.927	1.025	0.429

MACE (Ref.=Losartan)	Crude model				Adjusted model*				Adjusted model (with age variable)			
	HR	95% CI		P-value	HR	95% CI		P-value	HR	95% CI		P-value
		Low	High			Low	High			Low	High	
**Candesartan**	1.089	1.030	1.152	0.050	1.058	1.000	1.119	0.279	1.064	1.006	1.125	0.351
**Fimasartan**	1.062	0.958	1.178	0.613	1.078	0.972	1.195	0.313	1.098	0.990	1.218	0.223
**Irbesartan**	1.105	1.035	1.181	0.031	1.062	0.994	1.135	0.285	1.081	1.012	1.156	0.175
**Olmesartan**	1.017	0.968	1.068	0.341	1.018	0.969	1.070	0.602	1.028	0.979	1.080	0.612
**Telmisartan**	0.973	0.929	1.020	0.002	0.980	0.935	1.027	0.017	0.987	0.942	1.035	0.013
**Valsartan**	1.028	0.985	1.072	0.584	1.017	0.974	1.062	0.511	1.024	0.981	1.069	0.421

*The following variables were adjusted: sex, diabetes mellitus, dyslipidemia, smoking, drinking, exercise, body mass index, systolic blood pressure, albuminuria, glomerular filtration rate, and concomitant medications including calcium antagonists, beta-blockers, angiotensin converting enzyme inhibitor, diuretics, antithrombotic agents, and statins.

CI, confidence interval; HR, hazard ratio.

Supplementary **Table S4-2.** Results of **Cox-regression analyses showing the hazard ratios of mortality and MACE according to different ARBs in patients <70 years old (n=626,358)**

**All-cause mortality (Ref.=Losartan)**	**Crude model**				**Adjusted model***				**Adjusted model (with age variable)**			
	HR	95% CI		P-value	HR	95% CI		P-value	HR	95% CI		P-value
		Low	High			Low	High			Low	High	
**Candesartan**	0.956	0.882	1.037	0.216	0.968	0.893	1.050	0.360	1.002	0.924	1.086	0.438
**Fimasartan**	0.750	0.652	0.862	0.001	0.817	0.711	0.940	0.023	0.893	0.777	1.027	0.150
**Irbesartan**	1.001	0.908	1.103	0.035	0.958	0.869	1.056	0.603	0.976	0.885	1.076	0.976
**Olmesartan**	0.887	0.830	0.948	0.274	0.937	0.877	1.001	0.988	0.976	0.913	1.044	0.955
**Telmisartan**	0.889	0.837	0.945	0.270	0.932	0.876	0.990	0.817	0.970	0.913	1.032	0.866
**Valsartan**	0.953	0.900	1.010	0.111	0.961	0.907	1.018	0.331	1.010	0.953	1.070	0.162

**MACE (Ref.=Losartan)**	**Crude model**				**Adjusted model***				**Adjusted model (with age variable)**			
	HR	95% CI		P-value	HR	95% CI		P-value	HR	95% CI		P-value
		Low	High			Low	High			Low	High	
**Candesartan**	1.068	1.017	1.121	<0.001	1.047	0.997	1.099	0.008	1.071	1.020	1.125	0.016
**Fimasartan**	0.827	0.761	0.899	<0.001	0.889	0.817	0.966	0.003	0.950	0.873	1.032	0.052
**Irbesartan**	1.129	1.066	1.197	<0.001	1.066	1.006	1.130	0.003	1.079	1.018	1.143	0.022
**Olmesartan**	0.946	0.909	0.986	0.066	0.982	0.943	1.023	0.655	1.012	0.971	1.054	0.704
**Telmisartan**	0.926	0.892	0.962	0.001	0.971	0.934	1.008	0.225	1.002	0.964	1.040	0.307
**Valsartan**	0.975	0.941	1.011	0.885	0.986	0.951	1.022	0.780	1.022	0.986	1.060	0.810

*The following variables were adjusted: sex, diabetes mellitus, dyslipidemia, smoking, drinking, exercise, body mass index, systolic blood pressure, albuminuria, glomerular filtration rate, and concomitant medications including calcium antagonists, beta-blockers, angiotensin converting enzyme inhibitor, diuretics, antithrombotic agents, and statins.

CI, confidence interval; HR, hazard ratio.

Supplementary Table S4-3. Results of Cox-regression analyses showing the hazard ratios of mortality and MACE according to different ARBs in male patients (n=442,694)

**All-cause mortality (Ref.=Losartan)**	**Crude model**				**Adjusted model***			
	HR	95% CI		P-value	HR	95% CI		P-value
		Low	High			Low	High	
**Candesartan**	0.882	0.827	0.941	0.004	0.984	0.922	1.050	0.531
**Fimasartan**	0.677	0.603	0.760	0.0003	0.951	0.847	1.067	0.751
**Irbesartan**	0.893	0.826	0.967	0.006	0.928	0.858	1.005	0.251
**Olmesartan**	0.754	0.715	0.796	0.002	0.980	0.928	1.034	0.558
**Telmisartan**	0.726	0.690	0.762	<0.001	0.935	0.889	0.983	0.140
**Valsartan**	0.802	0.766	0.840	0.527	0.988	0.942	1.035	0.292

**MACE (Ref.=Losartan)**	**Crude model**				**Adjusted model***			
	HR	95% CI		P-value	HR	95% CI		P-value
		Low	High			Low	High	
**Candesartan**	1.049	0.997	1.103	<0.001	1.078	1.024	1.133	0.172
**Fimasartan**	0.827	0.757	0.902	0.0002	1.040	0.952	1.135	0.877
**Irbesartan**	1.135	1.069	1.204	<0.001	1.109	1.045	1.178	0.023
**Olmesartan**	0.886	0.849	0.924	<0.001	1.031	0.988	1.076	0.427
**Telmisartan**	0.882	0.849	0.917	<0.001	1.030	0.990	1.070	0.351
**Valsartan**	0.928	0.894	0.963	0.097	1.038	1.000	1.077	0.635

*The following variables were adjusted: age, diabetes mellitus, dyslipidemia, smoking, drinking, exercise, body mass index, systolic blood pressure, albuminuria, glomerular filtration rate, and concomitant medications including calcium antagonists, beta-blockers, angiotensin converting enzyme inhibitor, diuretics, antithrombotic agents, and statins.

CI, confidence interval; HR, hazard ratio.

Supplementary Table S4-4. Results of Cox-regression analyses showing the hazard ratios of mortality and MACE according to different ARBs in female patients (n=338,091)

All-cause mortality (Ref.=Losartan)	Crude model				Adjusted model*			
	HR	95% CI		P-value	HR	95% CI		P-value
		Low	High			Low	High	
**Candesartan**	0.922	0.846	1.004	0.136	1.013	0.930	1.104	0.910
**Fimasartan**	0.728	0.624	0.849	0.007	1.019	0.873	1.189	0.885
**Irbesartan**	0.921	0.830	1.022	0.226	0.980	0.883	1.087	0.515
**Olmesartan**	0.888	0.826	0.955	0.561	1.044	0.971	1.123	0.293
**Telmisartan**	0.808	0.753	0.866	0.015	1.021	0.951	1.095	0.718
**Valsartan**	0.861	0.807	0.919	0.675	0.989	0.927	1.055	0.486

MACE (Ref.=Losartan)	Crude model				Adjusted model*			
	HR	95% CI		P-value	HR	95% CI		P-value
		Low	High			Low	High	
**Candesartan**	1.031	0.977	1.087	<0.001	1.054	0.999	1.112	0.030
**Fimasartan**	0.794	0.721	0.875	<0.001	0.958	0.869	1.055	0.284
**Irbesartan**	1.058	0.992	1.128	<0.001	1.045	0.980	1.114	0.135
**Olmesartan**	0.931	0.888	0.975	0.609	1.004	0.958	1.052	0.912
**Telmisartan**	0.851	0.814	0.890	<0.001	0.952	0.910	0.996	0.010
**Valsartan**	0.948	0.910	0.987	0.671	1.004	0.964	1.046	0.891

*The following variables were adjusted: age, diabetes mellitus, dyslipidemia, smoking, drinking, exercise, body mass index, systolic blood pressure, albuminuria, glomerular filtration rate, and concomitant medications including calcium antagonists, beta-blockers, angiotensin converting enzyme inhibitor, diuretics, antithrombotic agents, and statins.

CI, confidence interval; HR, hazard ratio.

Supplementary Table S4-5. Results of Cox-regression analyses showing the hazard ratios of mortality and MACE according to different ARBs in diabetes patients (n=190,528)

**All-cause mortality (Ref.=Losartan)**	**Crude model**				**Adjusted model***			
	HR	95% CI		P-value	HR	95% CI		P-value
		Low	High			Low	High	
**Candesartan**	0.874	0.803	0.950	0.764	0.959	0.882	1.043	0.334
**Fimasartan**	0.871	0.749	1.013	0.833	1.103	0.948	1.283	0.112
**Irbesartan**	0.902	0.820	0.993	0.609	0.964	0.875	1.061	0.464
**Olmesartan**	0.838	0.779	0.902	0.103	0.980	0.910	1.055	0.664
**Telmisartan**	0.825	0.771	0.883	0.023	0.953	0.891	1.021	0.163
**Valsartan**	0.883	0.829	0.940	0.986	1.005	0.943	1.071	0.687

**MACE (Ref.=Losartan)**	**Crude model**				**Adjusted model***			
	HR	95% CI		P-value	HR	95% CI		P-value
		Low	High			Low	High	
**Candesartan**	1.009	0.947	1.075	0.051	1.055	0.990	1.124	0.293
**Fimasartan**	0.836	0.739	0.946	0.012	0.984	0.870	1.114	0.450
**Irbesartan**	1.011	0.940	1.088	0.078	1.026	0.954	1.104	0.966
**Olmesartan**	0.939	0.888	0.993	0.466	1.034	0.977	1.094	0.712
**Telmisartan**	0.926	0.879	0.975	0.152	1.021	0.969	1.075	0.859
**Valsartan**	0.985	0.938	1.034	0.163	1.056	1.005	1.108	0.168

*The following variables were adjusted: age, sex, dyslipidemia, smoking, drinking, exercise, body mass index, systolic blood pressure, albuminuria, glomerular filtration rate, and concomitant medications including calcium antagonists, beta-blockers, angiotensin converting enzyme inhibitor, diuretics, antithrombotic agents, and statins.

CI, confidence interval; HR, hazard ratio.

Supplementary Table S4-6. Results of Cox-regression analyses showing the hazard ratios of mortality and MACE according to different ARBs in non-diabetes patients (n=590,257)

All-cause mortality (Ref.=Losartan)	Crude model				Adjusted model*			
	HR	95% CI		P-value	HR	95% CI		P-value
		Low	High			Low	High	
**Candesartan**	0.885	0.829	0.945	0.003	1.020	0.955	1.089	0.151
**Fimasartan**	0.643	0.572	0.723	<0.001	0.916	0.815	1.029	0.194
**Irbesartan**	0.845	0.777	0.918	0.284	0.936	0.861	1.018	0.229
**Olmesartan**	0.803	0.761	0.847	0.647	1.019	0.966	1.075	0.088
**Telmisartan**	0.7440	0.707	0.782	<0.001	0.974	0.926	1.025	0.867
**Valsartan**	0.811	0.773	0.850	0.935	0.987	0.941	1.035	0.676

MACE (Ref.=Losartan)	Crude model				Adjusted model*			
	HR	95% CI		P-value	HR	95% CI		P-value
		Low	High			Low	High	
**Candesartan**	1.033	0.987	1.080	<0.001	1.073	1.026	1.123	0.027
**Fimasartan**	0.812	0.752	0.876	<0.001	1.007	0.933	1.087	0.547
**Irbesartan**	1.106	1.047	1.167	<0.001	1.115	1.056	1.178	0.001
**Olmesartan**	0.887	0.855	0.921	0.002	1.011	0.973	1.050	0.315
**Telmisartan**	0.838	0.809	0.869	<0.001	0.984	0.950	1.020	0.006
**Valsartan**	0.906	0.876	0.936	0.030	1.008	0.975	1.043	0.199

*The following variables were adjusted: age, sex, dyslipidemia, smoking, drinking, exercise, body mass index, systolic blood pressure, albuminuria, glomerular filtration rate, and concomitant medications including calcium antagonists, beta-blockers, angiotensin converting enzyme inhibitor, diuretics, antithrombotic agents, and statins.

CI, confidence interval; HR, hazard ratio.

Supplementary Table S5-1. Risks for all-cause mortality and MACE according to different ARBs compared to those associated with losartan (only ARB)

All-cause mortality (Ref.=Losartan)	Crude model				Adjusted model*			
	HR	95% CI		P-value	HR	95% CI		P-value
		Low	High			Low	High	
**Candesartan**	0.778	0.634	0.956	0.717	0.918	0.747	1.128	0.461
**Fimasartan**	0.694	0.514	0.938	0.262	1.024	0.757	1.384	0.744
**Irbesartan**	0.701	0.534	0.920	0.247	0.769	0.586	1.009	0.040
**Olmesartan**	0.834	0.721	0.966	0.568	1.083	0.936	1.254	0.128
**Telmisartan**	0.830	0.728	0.947	0.591	1.092	0.957	1.246	0.069
**Valsartan**	0.828	0.725	0.946	0.619	1.023	0.896	1.169	0.481

MACE (Ref.=Losartan)	Crude model				Adjusted model*			
	HR	95% CI		P-value	HR	95% CI		P-value
		Low	High			Low	High	
**Candesartan**	1.164	0.960	1.412	0.065	1.256	1.035	1.524	0.109
**Fimasartan**	0.940	0.702	1.258	0.627	1.127	0.842	1.510	0.843
**Irbesartan**	1.217	0.961	1.541	0.052	1.238	0.978	1.569	0.241
**Olmesartan**	1.001	0.860	1.165	0.978	1.138	0.977	1.326	0.596
**Telmisartan**	0.798	0.689	0.926	<0.001	0.925	0.797	1.074	0.006
**Valsartan**	0.934	0.811	1.077	0.266	1.053	0.913	1.214	0.473

*The following variables were controlled in the adjusted model: age, sex, body mass index, systolic blood pressure, diabetes mellitus, dyslipidemia, cigarette smoking, alcohol drinking, physical activity, household income, glomerular filtration rate.

MACE, major adverse cardiovascular event; ARB, angiotensin receptor blocker; HR, hazard ratio; CI, confidence interval

Supplementary Table S5-2. Risks for all-cause mortality and MACE according to different ARBs compared to those associated with losartan (ARB with one class of anti-hypertensive drug)

**All-cause mortality (Ref.=Losartan)**	**Crude model**				**Adjusted model***			
	HR	95% CI		P-value	HR	95% CI		P-value
		Low	High			Low	High	
**Candesartan**	0.808	0.712	0.916	0.913	0.999	0.880	1.134	0.988
**Fimasartan**	0.732	0.598	0.895	0.230	1.061	0.867	1.298	0.488
**Irbesartan**	0.823	0.705	0.961	0.848	0.949	0.812	1.109	0.456
**Olmesartan**	0.800	0.725	0.882	0.721	1.029	0.933	1.136	0.486
**Telmisartan**	0.739	0.674	0.811	0.022	0.955	0.870	1.048	0.282
**Valsartan**	0.813	0.745	0.888	0.977	0.999	0.915	1.091	0.975

**MACE (Ref.=Losartan)**	**Crude model**				**Adjusted model***			
	HR	95% CI		P-value	HR	95% CI		P-value
		Low	High			Low	High	
**Candesartan**	0.925	0.825	1.036	0.702	1.024	0.913	1.148	0.775
**Fimasartan**	0.901	0.757	1.073	0.930	1.095	0.919	1.304	0.285
**Irbesartan**	0.932	0.810	1.073	0.657	0.990	0.860	1.139	0.745
**Olmesartan**	0.907	0.829	0.991	0.986	1.024	0.936	1.120	0.712
**Telmisartan**	0.857	0.788	0.932	0.119	0.990	0.910	1.077	0.601
**Valsartan**	0.839	0.772	0.911	0.030	0.950	0.874	1.032	0.094

*The following variables were controlled in the adjusted model: age, sex, body mass index, systolic blood pressure, diabetes mellitus, dyslipidemia, cigarette smoking, alcohol drinking, physical activity, household income, glomerular filtration rate.

MACE, major adverse cardiovascular event; ARB, angiotensin receptor blocker; HR, hazard ratio; CI, confidence interval

Supplementary Table S5-3. Risks for all-cause mortality and MACE according to different ARBs compared to those associated with losartan (ARB with two more classes of anti-hypertensive drug)

All-cause mortality (Ref.=Losartan)	Crude model				Adjusted model*			
	HR	95% CI		P-value	HR	95% CI		P-value
		Low	High			Low	High	
**Candesartan**	0.914	0.862	0.969	0.055	0.998	0.941	1.059	0.582
**Fimasartan**	0.745	0.667	0.832	0.001	0.978	0.875	1.093	0.899
**Irbesartan**	0.910	0.847	0.977	0.145	0.957	0.891	1.027	0.366
**Olmesartan**	0.852	0.810	0.897	0.378	0.993	0.943	1.045	0.695
**Telmisartan**	0.823	0.785	0.864	0.011	0.966	0.920	1.013	0.382
**Valsartan**	0.864	0.826	0.903	0.740	0.998	0.954	1.043	0.488

MACE (Ref.=Losartan)	Crude model				Adjusted model*			
	HR	95% CI		P-value	HR	95% CI		P-value
		Low	High			Low	High	
**Candesartan**	1.017	0.977	1.058	0.012	1.079	1.037	1.122	0.145
**Fimasartan**	0.871	0.811	0.936	<0.001	1.027	0.956	1.104	0.448
**Irbesartan**	1.061	1.012	1.112	<0.001	1.096	1.046	1.149	0.045
**Olmesartan**	0.955	0.923	0.988	0.195	1.047	1.012	1.084	0.748
**Telmisartan**	0.950	0.920	0.981	0.081	1.052	1.019	1.086	0.979
**Valsartan**	0.972	0.944	1.002	0.927	1.067	1.035	1.099	0.297

*The following variables were controlled in the adjusted model: age, sex, body mass index, systolic blood pressure, diabetes mellitus, dyslipidemia, cigarette smoking, alcohol drinking, physical activity, household income, glomerular filtration rate.

MACE, major adverse cardiovascular event; ARB, angiotensin receptor blocker; HR, hazard ratio; CI, confidence interval
